# Co-expression Networks Identify DHX15 RNA Helicase as a B Cell Regulatory Factor

**DOI:** 10.3389/fimmu.2019.02903

**Published:** 2019-12-10

**Authors:** Thiago Detanico, Richard Virgen-Slane, Seth Steen-Fuentes, Wai W. Lin, Antje Rhode-Kurnow, Elizabeth Chappell, Ricardo G. Correa, Michael J. DiCandido, M. Lamine Mbow, Jun Li, Carl F. Ware

**Affiliations:** ^1^Infectious and Inflammatory Disease Center, Sanford Burnham Prebys Medical Discovery Institute, La Jolla, CA, United States; ^2^Department of Immunology & Respiratory Disease Research, Boehringer Ingelheim Pharmaceuticals, Inc., Ridgefield, CT, United States

**Keywords:** B cell, DHX15, RNA helicase, antibodies, Bayesian network, WGCNA

## Abstract

Genome-wide co-expression analysis is often used for annotating novel gene functions from high-dimensional data. Here, we developed an R package with a Shiny visualization app that creates immuno-networks from RNAseq data using a combination of Weighted Gene Co-expression Network Analysis (WGCNA), xCell immune cell signatures, and Bayesian Network Learning. Using a large publicly available RNAseq dataset we generated a Gene Module-Immune Cell (GMIC) network that predicted causal relationships between DEAH-box RNA helicase (DHX)15 and genes associated with humoral immunity, suggesting that DHX15 may regulate B cell fate. Deletion of DHX15 in mouse B cells led to impaired lymphocyte development, reduced peripheral B cell numbers, and dysregulated expression of genes linked to antibody-mediated immune responses similar to the genes predicted by the GMIC network. Moreover, antigen immunization of mice demonstrated that optimal primary IgG1 responses required DHX15. Intrinsic expression of DHX15 was necessary for proliferation and survival of activated of B cells. Altogether, these results support the use of co-expression networks to elucidate fundamental biological processes.

## Introduction

The technological advances in the “Omics” field generating high-dimensional datasets requires advanced mathematics and computational biology models (1). Analysis of these large “Omics” datasets through machine learning methods provides an important source for discovering biological processes. Such approaches are transforming the biological and medical sciences (2, 3). Machine learning methods available to the broader scientific community could accelerate novel biological insights. Here, we developed an R package with a Shiny visualization app that uses a machine learning strategy to simplify the analysis of large datasets yielding novel insight into immune function.

Two statistical tools commonly used for analysis of genome-wide expression data to predict gene function and disease association through gene-modules are Weighted Gene Co-expression Network Analysis (WGCNA) and Bayesian Network learning (4, 5). Both methods use sample to sample variation to generate co-expression networks, however, Bayesian Network learning searches for parent to child relationships from observational data by testing different possible combinations (5, 6). This can be used to infer causality at the cost of greater computer power given the high dimensionality of transcriptomics data. Functional predictions in co-expression networks are based on a guilty by association principle, in which genes with highly correlated expression patterns are likely to have functional relationships in pathways.

One of the biological disciplines that have gained extraordinary benefit from the Omics approach is the one that studies the immune system under healthy and disease conditions. Large high-dimensional studies of immune cells and immune responses have profoundly increased our understanding of the inner works of the immune system in normal and stressed situations (7–12). Although, substantial information has been harvested from these Omics studies there is consensus that the possibilities for discovery within these datasets remain fruitful.

Here, we used an immune-modified pipeline from Agrahari et al. (11) to predict causal relationships between co-expression modules and immune cell signatures from bulk RNA expression data. This workflow utilizes the power of WGCNA for compressing high-dimensional expression data into module eigengenes, which are merged with immune cell signature scores. In this format, Bayesian Network learning and inference can generate a Gene Module-Immune Cell (GMIC) network on a standard desktop computer. The generated GMIC network predicted a novel function for DHX15, a member of the DExD/H-box RNA helicase family, in adaptive immune responses. Further *in vivo* and *in vitro* work uncovered a role for DHX15 in lymphocyte development and during humoral immune responses.

## Materials and Methods

### Gene-Module Immune Cell Network

RNAseq data from Diffuse Large B Cell Lymphoma patient biopsies (*n* = 562) were obtained (provided as normalized FPKM values in log_2_ scale) (12) and processed for analysis. To remove genes of low frequency, transcripts with 0 values in more than 90% of the samples were excluded. The expression profile of a subset of patients (*n* = 5) were excluded as outliers based on sample clustering. A final matrix containing 21,565 transcripts for 557 patients was analyzed using the WGCNA package in the R statistical computing environment (13). A “signed hybrid” network was generated using the “bicor” setting, soft threshold power of five, and a minimum module size of 10. Modules with distance heights lower than 0.25 were merged, which resulted in 69 modules. Names for modules were generated based on gene ontology enrichment using the GOstats package in R (14). Cell signature scores for patients were generated from the processed expression matrix using xCell (15) with default settings.

To infer causal relationships between modules and cell signature scores, we used the bnlearn package in R (16). Briefly, cell signature scores (centered and scaled) were merged with module eigengenes and then discretized into three breaks using Hartemink's method (17). The Bayesian Network learning was carried out using the boot.strength function (500 replicates) with default tabu settings and bde score. Networks were averaged using the averaged.network function with default settings.

### Code Availability

We developed the GMIC generating code into an R package, called GmicR, which can be download from Bioconductor via doi: 10.18129/B9.bioc.GmicR.

### Computer

GMIC network was performed on an iMac 4 GHz intel Core i7 processor with 32 GB of RAM. The total computer running time was approximately 1.3 days for the complete immune-network.

### Mice

*Dhx15*^*flox*^ ES cell line in the C57Bl/6 background was obtained from EUCOMM, and injection of pseudo-pregnant females was performed by the Mouse Genetic Core at The Scripps Research Institute (La Jolla, CA). Briefly, a construct containing the *Dhx15* exon2 sequence flanked by two flox sites was used for the generation of the targeted knock-in in JM8A3.N1 ES cell line. Neomycin resistant ES clones were selected and screened for locus-specific target insertion by PCR, and positive ES clones were selected for *in vivo* injections. Neomycin resistant gene was excised by crossing *Dhx15*^*flox*^ mice to a *B6.FLPo* expressing strain. *Dhx15*^*flox*/*flox*^ mice were maintained in house as either homozygous or crossed to *Cd19*^*cre*^ and *Cd4*^*Cre*^. The *B6.129P2(C)-Cd19*^*tm*1(*cre*)*Cgn*^*/J* (*Cd19*^*cre*^) mouse was provided by Rickert et al. (18). *B6.SJL-PtprcaPepcb/BoyJ (B6.SJL)* and *B6.Cg-Tg(Pgk1-flpo)10Sykr/J* (*B6.FLPo*) (19) mice were purchased from Jackson Laboratory (Bar Harbor, ME). Validation of DHX15 knock-out is shown in Supplementary Figure 2. All mice were backcrossed and housed in the Animal Facility at Sanford Burnham Prebys Medical Discovery Institute (La Jolla, CA), and experiments were conducted with the approval of the Institute's IACUC ethics committee.

### DNA Extraction and PCR Genotyping

Extraction of DNA from mouse tails used the QuickExtract DNA Extraction (Lucigen), following standard manufacture's procedure. Amplification of *Dhx15* was carried out using a Biorad C1000 thermal cycler with a programmed cycle of 3 min for the initial denaturation at 95°C, 35 cycles of 30 s at 95°C for denaturation, 30 s at 61°C for annealing, and 30 s at 72°C for extension with the final extension at 72°C for 3 min. PCR reactions used for *Cd19*^*cre*^ and *Cd4*^*cre*^ genotyping were, respectively; 2 min of initial denaturation at 94°C; 35 cycles of 1 min at 94°C for denaturation, 1 min at 62°C for annealing, and 1.5 min at 72°C for extension; final extension at 72°C for 5 min; *CD4*^*cr*^^e^ for 4 min of initial denaturation at 94°C; 35 cycles of 30 s at 94°C for denaturation, 45 s at 60°C for annealing, and 45 s at 72°C for extension with final extension at 72°C for 5 min. The list of primers is provided in Supplementary Table 2.

### Immunization and IgG1 ELISA

Mice were immunized with chicken gamma globulin conjugated with the hapten 4-hydroxy-3-nitrophenylacetyl (NP-CGG, Biosearch Technologies), 100 μg/ml in saline was mixed at 1:1(v/v) ratio with the adjuvant Imject® Alum (Thermo Scientific), and mice immunized by intraperitoneal injection. Mice were sedated with isoflurane prior to retro-orbital blood collection. Specific antibodies (Ab) against NP were determined by ELISA, using NP_18_-BSA (Biosearch Technologies) and SBA Clonotyping System-C57BL/6-HRP (SouthernBiotech) as previously described (20). NP Specific Abs to the NP hapten were determined by 2–3-fold serial dilution of serum samples, and relative anti-NP Ab units were calculated using a standard serum pool from mice immunized with NP-CGG.

### RNA Extraction, cDNA Synthesis, and RT-qPCR

RNA was extracted using RNeasy Mini kit (QIAGEN) and quantified using the ThermoFisher Nanodrop One. cDNA was synthesized using the iScript™ cDNA Synthesis Kit (BioRad) and RT-qPCR used the iTaq^TM^ Universal SYBR Green One-Step Kit (primers listed in Supplementary Table 2).

### NanoString

NanoString nCounter® Mouse Immunology Panel assay was carried following manufacturer's instructions. Data was analyzed using the DESeq2 (21) package for R (Wald's Test), with *apeglm* log-fold change shrinkage https://doi.org/10.1093/bioinformatics/bty895 and the following hypothesis model: (Raw NanoString data) ~ Genotype ^*^ Stimulation. P adjusted values (pAdj) were calculated using the Benjamini & Hochberg method. Differentially expressed genes with pAdj < 0.1 were considered significant.

### Flow Cytometry and Cell Culture

Animals were humanely euthanized by CO_2_ inhalation following IACUC approved standard procedures. Organs were harvested and single cell suspensions generated using a 70 μm cell strainer (Corning). Cell suspensions were pre-treated with ACK red blood cell lysis buffer. Immunophenotyping was performed in the presence of PBS supplemented with 2% FCS and 0.05% azide on ice. Analytical cytometry was performed in the Sanford Burnham Prebys Medical Discovery Institute Flow Cytometry Core. B cells were purified with EasySep B cell enrichment kit (STEMCELL Technologies), and stimulation assays were performed as indicated in the figure legends using mouse BAFF (25 ng/ml), anti-CD40 (5 μg/ml), and anti-IgM (10 μg/ml). Supplementary Table 2 provides a list of the reagents used.

### Statistics

*P* values were calculated using the multiple linear regression function on R studio. Graphs were generated using GraphPad Prism version 8.0.0 (San Diego, CA).

## Results

### Co-expression Analysis and Gene-Module Immune Cell Network

**Figure 1:**
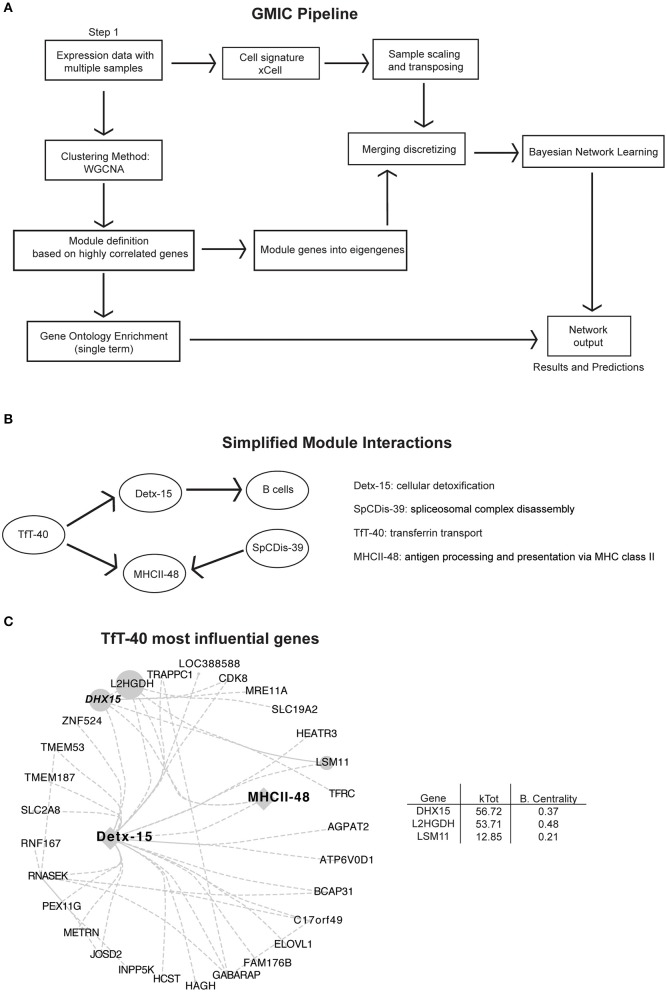
Analysis pipeline and immune-networks. RNAseq data from 557 subjects originally described by Schmitz et al. (12) was used in our co-expression workflow to create the modular immune-network. **(A)** Schematic illustration of the analysis pipeline used to generate the immune-networks. Cell signatures were added using xCell signature-based method, and co-expression genes were first determined using WGCNA. Eigengenes were determined using principal component analysis of the modular genes. Bayesian Network learning was applied to eigengenes to create the full immune-network and modules with cell signature. An open access version of the GMIC generating package is available from doi: 10.18129/B9.bioc.GmicR. **(B)** Simplified modular network from Supplementary Figure 1A, focused on MHCII class II presentation (MHCII-48), and module TfT-40. **(C)** Representation of the most influential genes for the TfT-40 module. The total number of edges were minimized by using a 0.04 threshold. Sizes of nodes and labels represent betweenness centrality calculated by Cytoscape from the depicted directed network, and it is proportional to the gene influence. DHX15 is highlighted in a bold italic font. For an expanded version see Supplementary Figure 1B.

The current advances in computational biology and machine learning have opened the possibilities to retroactively investigate and re-purpose public large datasets to generate hypotheses based on novel gene-gene co-expression relationships. Here, we incorporated an immune cell signature algorithm to the co-expression workflow from Agrahari et al. (11) to develop an open access Shiny visualization app for the analysis and the generation of GMIC networks from expression datasets. As a proof of concept, we generated a GMIC network from a publicly available large RNA expression dataset from lymphoma patient biopsies. We chose this dataset because it was readily accessible, had a high sample count (*n* = 557), and contains heterogeneous molecular signatures. Although, B cells are the predominant cell type from these biopsies, we used xCell to enumerate other detectable immune cell signatures. In this immune-network, genes with similar co-expression patterns were segregated into 70 gene-modules (69 functional modules plus module 0) (Supplementary Figure 1A). By including immune-cell signature scores and emphasizing biological function, rather than predicting markers of disease, we were able to infer causality between immune cell signatures and eigengenes (Figure 1A). This workflow was used to investigate immune-pathways and to uncover new gene-gene relationships from a published RNAseq dataset (12), as shown in Figure 1B (see Supplementary Figure 1A and Supplementary Table 1 for a complete list of genes and modules). An open access version of the GMIC generating package is available for download at doi: 10.18129/B9.bioc.GmicR.

We focused our analysis on modules linked to antigen presentation, with particular emphasis on the MHC class II pathway module (MHCII-48). Antigen presentation of exogenous antigens via MHC II is one of the pillars of the adaptive immune response, and this pathway is essential for both healthy and diseased immune responses (22). The GMIC network predicted that transferrin transport (TfT-40) and spliceosomal complex disassembly (SpCDis-39) had a strong relationship with the MHC-II module. Moreover, TfT-40 module could indirectly influence the B cell signature in the samples, through its relationship with cellular detoxification module 15 (Detx-15) (Figure 1B and Supplementary Figure 1). Among the genes in TfT-40, DHX15 stood out as a candidate because of its rank of influence on MHCII-48, TfT-40, and Detx-15 modules (highest kTotal with the second highest between-centrality score, Figure 1C and Supplementary Figure 1B) as well as its known function as an innate immune sensor (23–25).

### Lymphopenia in Conditional *Dhx15* Deficient Mice

**Figure 2:**
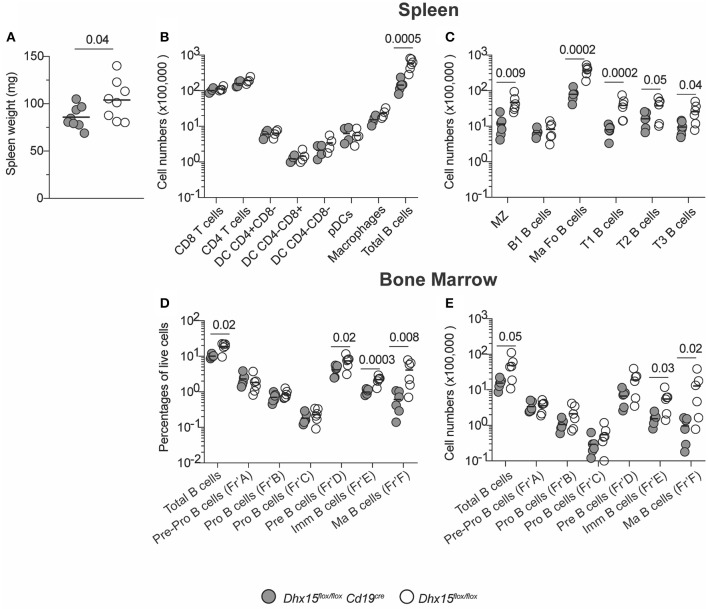
B cell lymphopenia in DHX15 B cell conditional KO mice. **(A)** Spleen weight in mg from experimental (filled symbols) and negative littermates (opened symbols). **(B)** Immunophenotyping of the spleen. Cell numbers were defined by the percentages of CD45^+^Singlets^+^Live gate^+^ (Total cells). Additional leukocyte gates were defined as followed: T cells (CD3^+^B220^−^CD19^−^), B cells (B220^+^CD19^+^CD3^−^), DCs (CD11c^+^CD19^−^CD3^−^F4/80^−^), pDCs (DCgate^+^CD11c^int^B220^+^), and macrophages (F4/80^+^CD19^−^CD3^−^CD11c^−^). **(C)** Spleen B cell populations were defined according to Supplementary Figure 3A. **(D,E)** Bone marrow immunophenotyping. B cell development fractions were determined using Supplementary Figure 3B gating strategy. Percentages and total numbers were relative to the Live gate^+^ Singlets^+^ FSC-A/SSC-A gate. Total bone marrow cell numbers were relative to two fibulas per mouse. Each symbol represents an individual animal, from the combined results of 3–4 experiments. Animals were 8–14 weeks of age, and from both sexes. Statistical analysis was performed with R studio using the multiple linear regression function and the following equation: rank(Variable Y)~Genotype + Sex + Replicate. Only *P* values smaller than 0.05 were reported.

The significant influence of DHX15 on module TfT-40, as well as the direct-relationship of TfT-40 and Detx-15, and consequently the B cell signature in our analysis, suggested a novel and consequential role for DHX15 in modulating humoral immune responses. Understanding the role of DHX15 in primary human B cells faced technical challenges including limited cell numbers due to donor availability and difficulty to manipulate genetically without use of an activating stimulus. The high degree of similarity between human and mouse DHX15 RNA sequences (>92%), prompted the development of a mouse strain in which DHX15-deficiency can be restricted to the B cell lineage (Supplementary Figure 2). Ablation of *Dhx15*^*flox*/*flox*^ driven by the *Cd19* promoter resulted in mice with decreased splenic mass and cellularity (Figures 2A,B). The *Dhx15*^*flox*/*flox*^*Cd19*^*cre*^ mice showed no differences in all major leukocyte cell types, except for an approximate 4-fold reduction in the total numbers of B220^+^CD19^+^ B cells (Figure 2B). Curiously, B cell lymphopenia was not restricted to a particular B cell subtype (Figure 2C), suggesting that B cell development was impaired in *Dhx15*^*flox*/*flox*^*Cd19*^*cre*^ mice. To test this hypothesis, we analyzed bone marrow cells from *Dhx15*^*flox*/*flox*^*Cd19*^*cre*^ and littermate control mice. Indeed, defects in B cell development occurred as early as the pre-B cell stage in *Dhx15*^*flox*/*flox*^ *Cd19*^*cre*^ mice (Figures 2D,E).

**Figure 3:**
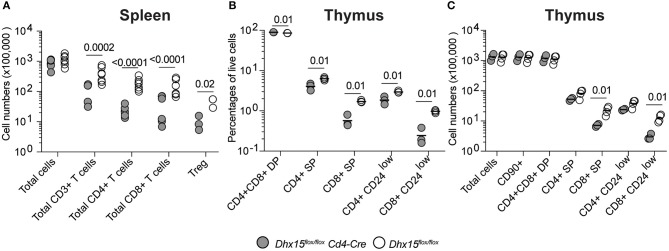
T cell deficiency and impaired thymocyte development in *Dhx15*^*flox*/*flox*^ *Cd4-Cre* mice. **(A)** Total cell counts (total CD45^+^ cells) and T cell subtypes from *Dhx15*^*flox*/*flox*^ *Cd4-Cre*^+^(filled symbols) and *Dhx15*^*flox*/*flox*^ negative littermate (opened symbols). Cell gates were defined as in Figure 2. Treg cells were defined by the CD4^+^ T cell scheme gate followed by segregation using the Foxp3 stain. **(B,C)** Immunophenotyping of the thymus. Cell numbers were calculated using a CD45^+^Singlets^+^Live gate^+^. Each symbol represents an individual animal, from the combined results of 2 experiments. Animals were 8–14 weeks of age, and from both sexes. Statistical analysis was performed with R studio using the multiple linear regression function and the following equation: rank(Variable Y)~Genotype + Sex + Replicate. Only *P* values smaller than 0.05 were shown.

DHX15 is broadly expressed in hematopoietic cells (Immunological Genome Project database (26), http://www.immgen.org/), and some members of the DExD/H-box RNA helicase family are known to be essential for RNA metabolism (27–29), suggesting that DHX15 function in hematopoiesis extends beyond the B lymphocyte lineage. To determine if the role of DHX15 in hematopoiesis is restricted to the B cell compartment, we crossed the *Dhx15*^*flox*/*flox*^ mice to a *Cd4-Cre* expressing strain, specifically ablating DHX15 expression in T cells. In this Cre-recombinase expressing model, conditional deletion occurs in the thymus at the double-positive stage during thymocyte development, and both mature CD4 and CD8 single-positive cells are equally affected (30).Deletion of *Dhx15* in T cells resulted in an approximately 4-fold reduction in the total numbers of CD3^+^ splenic T cells (Figure 3A), indicating a function for DHX15 during T cell development. In confirmation, both CD4^+^ and CD8^+^ single positive thymocytes were reduced in *Dhx15*^*flox*/*flox*^*Cd4*^*cre*^ mice when compared to control mice (Figures 3B,C). Together these results demonstrate a requirement for DHX15 in T and B cell development.

### DHX15 Modulates the Expression of Several Genes Linked to Antibody Responses

**Figure 4:**
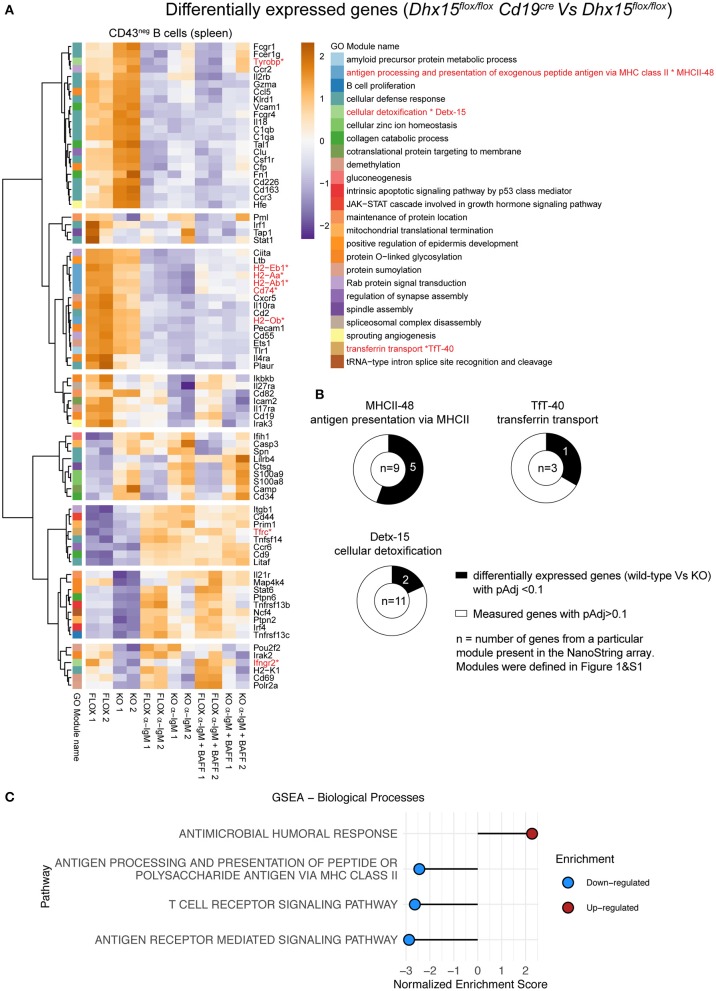
Differential gene expression in DHX15 deficient B cells. NanoString analysis of negative selected purified B cells from the spleens of *Dhx15*^*flox*/*flox*^ *Cd19*^*cre*^ and *Dhx15*^*flox*/*flox*^ mice. Cultured B cells were stimulated or not with anti-IgM ± BAFF for 22 h, followed by RNA extraction. NanoString data was normalized using DESeq2. **(A)** A total of 98 genes were significantly differentially expressed (pAdj < 0.1) between DHX15-null B cells and controls. Significant genes were matched to the human WGCNA gene modules as described in Supplementary Table 1, and differentially expressed genes belonging to module 0 were excluded from the heatmap (total of 17 genes). Expression levels represent DESeq2-normalized values, scaled by row. Color-coding on the left represents individual WGCNA gene modules as annotated on the right and defined in Figure 1 and Supplementary Figure 1. *Represent the differentially expressed genes that belong to modules MHCII-48, TfT-40, and Detx-15. **(B)** Doughnut graphs represent the total number of genes measured by NanoString that belong to MHCII-48, TfT-40, and Detx-15. **(C)** Gene set enrichment analysis of differentially expressed genes, using the GO gene set (biological processes) from the Molecular Signatures Database. Top 4 pathways are shown, which were identified from genes differentially expressed in DHX15-null B cells.

The relationships predicted by our computational analysis between Detx-15 and B cells, Detx-15 and TfT-40 (DHX15 module), and TfT-40 and MHCII-48, suggested that DHX15 may function outside lymphopoiesis, in particular during Ab immune responses. To investigate the role of DHX15 during immune responses, we used NanoString to analyze gene expression in splenic B cells from *Dhx15*^*flox*/*flox*^ *Cd19*^*cre*^ mice and *Dhx15*^*flox*/*flox*^ littermate controls (Figure 4). RNA expression analysis revealed that several genes are differentially expressed between control and experimental groups, under unstimulated conditions or following co-activation of the antigen receptor and BAFFR or CD40. Approximately 18% of the genes tested on the NanoString Immunology array panel showed differential expression (98 genes of 560 genes measured on the array) (Figure 4A), and among these genes, some were independent of Dhx15 deficiency, and likely due to differences in the *Cd19* locus between the experimental and control groups used in this assay (Supplementary Figures 4A–D). To circumvent this caveat, we focused on orthologous genes that were part of the modules that had a direct link with DHX15 (MHCII-48, TfT-40, and Detx-15). As predicted by the GMIC network data (Figure 1 and Supplementary Figure 1), a high frequency of the mouse orthologous genes tested from MHCII-48 (~56%), TfT-40 (~33%), and Detx-15 (~18%) modules were differentially expressed in DHX15-null B cells. The combined frequency of modular-genes differentially expressed in DHX15 null B cells were enriched approximately 2-fold relative to all differentially expressed genes (8 out of 23 vs. 98 out of 560 measured) (Figure 4B).

### Optimal Primary Antibody Responses Require DHX15 Expression in B Cells

**Figure 5:**
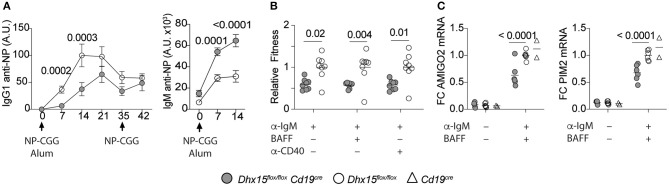
Reduced primary antibody responses in *Dhx15*^*flox*/*flox*^ *Cd19*^*cre*^ mice. **(A)** IgG1 and IgM anti-NP_18_BSA Abs in sera of mice immunized with NP-CGG in alum. Arrows indicated the immunization days. X-axis represent the time points when sera were collected. Arbitrary units (A.U.) were defined using pooled serum from animals that were immunized with NP-CGG. The graph represents the mean ± SEM from two independent experiments. Filled symbol represents the mean A.U. of IgG1 or IgM anti-NP Abs from *Dhx15*^*flox*/*flox*^ *Cd19*^*cre*^ (*n* = 12). Opened symbol represents the mean A.U. for *Dhx15*^*flox*/*flox*^ mice (*n* = 15). **(B)** Competitive fitness was determined by using a mixed co-cultured system of CD45.2^+^ and CD45.1^+^ splenocytes (CD45.2^+^ *Dhx15*^*flox*/*flox*^ *Cd19*^*cre*^ or CD45.2^+^ *Dhx15*^*flox*/*flox*^ with CD45.1^+^ wild-type cells). Relative Fitness was calculated after 72 h of *in vitro* stimulation with the indicated treatments by the following equation: [72 h Treatment Ratio (%B220^+^CD45.2^+^/%B220^+^CD45.1^+^)]:[0 h Ratio(%B220^+^CD45.2^+^/%B220^+^CD45.1^+^)]. Data represents the summary of two combined experiments. Each symbol represents an individual mouse. **(C)** Relative expression of PIM2 and AMIGO2 by RT-qPCR after 24 h stimulation with anti-IgM plus BAFF on purified B cells. Data represents the summary of three independent experiments, with *n* = 2 per experiment, except for the *Cd19*^*cre*^ animals (one experiment). Expression was normalized using ACTIN, RPL1a, and L32 genes. Fold-changes (FC) were calculated by dividing each data point by the mean normalized expression of the stimulated *Dhx15*^*flox*/*flox*^ group in each experiment. Each symbol represents an individual mouse. Animals were 8–14 weeks of age, and from both sexes. Statistical analysis was performed with R studio using multiple linear regression function and the following equations: **(A)** rank(A.U.)~Genotype*Bleed Day + Sex. **(B,C)** rank(Variable Y)~Genotype + Sex + Replicate. Only *P* values smaller than 0.05 were reported.

Gene set enrichment analysis of the differently expressed genes in DHX15-deficient B cells identified GO pathways known to directly or indirectly play a role in T cell-dependent Ab responses (31–37), by modulating antigen presentation and lymphocyte receptor signaling (Figure 4C). To investigate the requirements for DHX15 during T cell-dependent Ab-responses, we immunized DHX15 B cell deficient mice with 4-hydroxy-3-nitrophenylacetyl (NP) coupled to chicken gamma globulin (NP-CGG). DHX15 deficiency in B cells significantly impaired the early IgG1 anti-NP response in mice (Figure 5A). However, no difference in the specific IgG1 anti-NP memory response was observed (day 42) after a second immunization (Figure 5A). Interestingly, DHX15 deficiency enhanced the IgM response (Figure 5A), indicating that differences in the anti-NP IgG1 response was not merely a consequence of a lower frequency of NP-specific B cell clones in *Dhx15*^*flox*/*flox*^*Cd19*^*cre*^ mice.

### DHX15 Is Required for *in vitro* B Cell Responses

Tyrosine-Based Activation Motif-Bearing Adapter Protein (TYROBP; Detx-15) and Transferrin Receptor (TFRC; MHCII-48) were previously shown to be essential for B lymphocyte proliferation *in vivo* and *in vitro*. For example, lymphocyte deficiency in iron transport due to a polymorphism in the human TFRC gene, is linked to a severe immunodeficiency phenotype with a low proliferative response *in vitro* to mitogen stimulation (35). On the other hand, TYROBP, also known as DAP12, negatively regulates B cell proliferation, and TYROBP deficiency in B cells increases lymphocyte proliferation *in vitro* (34). Analysis of DHX15-null B cells showed enhanced TYROBP expression but decreased TFRC levels (Figure 4A and Supplementary Figure 4), supporting the model that DHX15 directly modulates B cell fate upon BCR stimulation. A similar correlation expression pattern for DHX15 was observed for the human lymphoma dataset as well as for a smaller lymphocyte dataset from healthy donors (Supplementary Figures 4E,F and Supplementary Table 3).

Given the previous results, we hypothesized that the decrease in the primary IgG1 response in *Dhx15*^*flox*/*flox*^*Cd19*^*cre*^ mice, occurs at least in part, as a consequence of suboptimal proliferation, or survival, or both. To determine the potential role of DHX15 in B cell proliferation and survival, independent of antigen presentation, we used a competitive fitness assay for B cells. As shown in Figure 5B, DHX15-null B cells decreased in relative proliferation and survival upon activation of the B cell antigen receptor (F(ab′)2 goat anti-mouse IgM) or inclusion of costimulating cytokines (BAFF or anti-CD40). Moreover, mRNA expression of PIM2 and AMIGO2, two genes previously linked to BAFF dependent B cell proliferation and survival (38), decreased in DHX15-null B cells when compared to controls (~1.6-fold for AMIGO2, and ~1.5-fold for PIM2). Combined, the data corroborates our *in silico* strategy identifying novel immune-associated functions for DHX15.

## Discussion

The use of co-expression tools in the investigation of high-dimensional data provides an important resource for understanding fundamental biological mechanisms. Here, we use an immune centric workflow analysis for large expression data that sequentially combines two co-expression analysis methods with xCell signature algorithm and GOstats to generate a GMIC network. This *in silico* model predicted a novel function for DHX15 during B cell-dependent immune responses by influencing modules containing MHC class II-associated genes, TYROBP and TFRC. *In vivo* and *in vitro* experiments with DHX15-deficient B cells confirmed several predictions of the GMIC model as well as demonstrated a function for DHX15 in lymphopoiesis and during primary Ab responses.

The GmicR pipeline condenses high-dimension Omics data into a format that allows for Bayesian Network learning and inference using a standard desktop computer. The compression of variables permits increased number of samples, thereby enhancing detection of relationships (11). With gene ontology enrichment, modules can be assigned biological functions, which then provide a rich platform for generating hypotheses. However, there are several limitations to our pipeline. First, to reduce the high-dimensionality of large data, Bayesian Network learning is carried out with module eigengenes. The benefit of the modular eigengenes is the reduction in the number of variables, while maintaining the observation count. For the lymphoma data analyzed in this study (*n* = 557), 21,565 genes were compressed into 69 eigengenes (69 functional modules plus a module 0). As a result, causality is not at the gene level, explaining why gene-modules containing some known B cell lineage factors were not observed directly influencing the B cell signature. However, since module functions are influenced within the module connections, it is reasonable to explore causality with experiments investigating genes with high influence on modules. This is the strategy we used to select DHX15 for study. Our pipeline uses WGCNA for module detection. Genes that are not assigned to a module, due to sample noise and parameter stringency, are grouped into module 0 and are left out of the analysis. According to one study, this subtraction of genes allows the use of noisy datasets, although leading to a loss of module information (4). Parameters for module detection do not change the relationships between individual genes, but they may influence some relationships in the global GMIC network. A similar outcome was observed when we performed our GmicR pipeline on a smaller dataset from healthy donors. Additionally, networks constructed using different datasets may also yield some differences in GMIC relationships. Ultimately, experimental validation of the predictions must be established. Finally, our workflow requires a large number of observations for learning. Bayesian Network learning works by generating multiple bootstrap replicates from random sampling in order to test the strength between nodes, hence the use of datasets with limited sample size may yield networks with minimum connections. In this situation, it may be best to pool datasets to increase the number of observations, which will enhance the detection of gene-gene relationships.

The generated GMIC network led us to DHX15, a protein belonging to the DExD/H-box RNA helicase superfamily known for having roles in RNA biology and in the intracellular recognition of viral nucleic acids (23–25). Our immune-network model predicted a novel function for DHX15. The human RNAseq dataset subjected to the co-expression pipeline inferred a close relationship of DHX15 with TFRC, TYROBP and MHCII-associated genes, and *in vitro* studies with mouse B cells supported this computational approach. Genes such as TFRC, that positively regulate antibody responses (31, 35, 39), showed reduced expression in DHX15-null B cells. It is possible that reduced expression of this key gene might be due to inefficient RNA biogenesis in DHX15-deficient B cells. However, this does not directly explain the increased expression levels of TYROBP observed in DHX15 deficient B cells. TYROBP is thought to be a negative regulator of B cell activation via recruitment of SHP1 phosphatase to the B cell receptor synapse (34). Expression data from DHX15 deficient B cells showed elevated TYROBP suggesting an indirect modulation of transcriptional processes by DHX15. Interestingly, Inesta-Vaquera et al. (40) recently reported that DHX15 forms a complex with CMRT1, an interferon-stimulated gene (ISG95) encoding a O-2 ribose methyltransferase involved in mRNA capping. The helicase function of DHX15 is activated by CMTRI, whereas DHX15 reciprocally reduces the enzymatic activity of CMTR1 O-2 methyltransferase activity. Disruption CMTR1-DHX15 complex affects selective sets of mRNAs involved in key metabolic functions and cell proliferation, and to a first degree phenocopy the cell proliferation and survival features we observe in DHX15-deficient mice. Thus, DHX15 may play a key role in innate immune recognition.

Overall, the experiments presented here support the use of co-expression networks to identify novel immune gene functions from expression Omics data.

## Data Availability Statement

The raw data supporting the conclusions of this manuscript will be made available by the authors, without undue reservation, to any qualified researcher.

## Ethics Statement

The animal study was reviewed and approved by Sanford Burnham Prebys Medical Discovery Institute, La Jolla, California 92037.

## Author Contributions

TD and RV-S: conceptualization, experimental design, data curation, analysis, validation, and writing. SS-F, WL, RC, MD, AR-K, and EC: data acquisition and validation. MM and JL: conceptualization, resources, and data analysis. CW: conceptualization, resources, data curation, project administration, and writing.

### Conflict of Interest

The authors disclose support for this project was provided in part by Boehringer Ingelheim with a research contract to Sanford Burnham Prebys Medical Discovery Institute. CW served as Principal Investigator of the contract and has no other potential conflict of interest with Boehringer Ingelheim; MM, JL, and MD were employees of Boehringer Ingelheim. The funder had the following involvement with the study: experimental design, data acquisition, and analysis. The remaining authors declare that the research was conducted in the absence of any commercial or financial relationships that could be construed as a potential conflict of interest.

## References

[1] Crouser ED Fingerlin TE Yang IV Maier LA Nana-Sinkam P Collman RG. Application of “Omics” and systems biology to sarcoidosis research. Ann Am Thorac Soc. (2017) 14:S445–51. 10.1513/AnnalsATS.201707-567OT 29053026 PMC5822413

[2] Ching T Himmelstein DS Beaulieu-Jones BK Kalinin AA Do BT Way GP. Opportunities and obstacles for deep learning in biology and medicine. J R Soc Interface. (2018) 15:20170387. 10.1098/rsif.2017.0387 29618526 PMC5938574

[3] Deo RC. Machine learning in medicine. Circulation. (2015) 132:1920–30. 10.1161/CIRCULATIONAHA.115.001593 26572668 PMC5831252

[4] Saelens W Cannoodt R Saeys Y A comprehensive evaluation of module detection methods for gene expression data. Nat Commun. (2018) 9:1090 10.1038/s41467-018-03424-4 29545622 PMC5854612

[5] van Dam S Vosa U van der Graaf A Franke L de Magalhaes JP. Gene co-expression analysis for functional classification and gene-disease predictions. Brief Bioinform. (2018) 19:575–92. 10.1093/bib/bbw139 28077403 PMC6054162

[6] Friedman N Linial M Nachman I Pe'er D. Using Bayesian networks to analyze expression data. J Comput Biol. (2000) 7:601–20. 10.1089/106652700750050961 11108481

[7] Ghosh D Bernstein JA Khurana Hershey GK Rothenberg ME Mersha TB. Leveraging multilayered “Omics” data for atopic dermatitis: a road map to precision medicine. Front Immunol. (2018) 9:2727. 10.3389/fimmu.2018.02727 30631320 PMC6315155

[8] Brown LV Gaffney EA Wagg J Coles MC. Applications of mechanistic modelling to clinical and experimental immunology: an emerging technology to accelerate immunotherapeutic discovery and development. Clin Exp Immunol. (2018) 193:284–92. 10.1111/cei.13182 30240512 PMC6150250

[9] Marty Pyke R Thompson WK Salem RM Font-Burgada J Zanetti M Carter H Evolutionary pressure against MHC class II binding cancer mutations. Cell. (2018) 175:416–28.e13. 10.1016/j.cell.2018.08.048 30245014 PMC6482006

[10] Chihara N Madi A Kondo T Zhang H Acharya N Singer M. Induction and transcriptional regulation of the co-inhibitory gene module in T cells. Nature. (2018) 558:454–9. 10.1038/s41586-018-0206-z 29899446 PMC6130914

[11] Agrahari R Foroushani A Docking TR Chang L Duns G Hudoba M. Applications of Bayesian network models in predicting types of hematological malignancies. Sci Rep. (2018) 8:6951. 10.1038/s41598-018-24758-5 29725024 PMC5934387

[12] Schmitz R Wright GW Huang DW Johnson CA Phelan JD Wang JQ. Genetics and pathogenesis of diffuse large B-cell lymphoma. N Engl J Med. (2018) 378:1396–407. 10.1056/NEJMoa1801445 29641966 PMC6010183

[13] Langfelder P Horvath S. WGCNA: an R package for weighted correlation network analysis. BMC Bioinformatics. (2008) 9:559. 10.1186/1471-2105-9-559 19114008 PMC2631488

[14] Falcon S Gentleman R. Using GOstats to test gene lists for GO term association. Bioinformatics. (2007) 23:257–8. 10.1093/bioinformatics/btl567 17098774

[15] Aran D Hu Z Butte AJ. xCell: digitally portraying the tissue cellular heterogeneity landscape. Genome Biol. (2017) 18:220. 10.1186/s13059-017-1349-1 29141660 PMC5688663

[16] Scutari M Learning Bayesian networks with the bnlearn R package. J Stat Softw. (2010) 35:1–22. 10.18637/jss.v035.i0321603108

[17] Hartemink AJ Principled Computational Methods for the Validation Discovery of Genetic Regulatory Networks. (Cambridge, MA: Massachusetts Institute of Technology) (2001).

[18] Rickert RC Roes J Rajewsky K. B lymphocyte-specific, Cre-mediated mutagenesis in mice. Nucleic Acids Res. (1997) 25:1317–8. 10.1093/nar/25.6.1317 9092650 PMC146582

[19] Wu Y Wang C Sun H LeRoith D Yakar S. High-efficient FLPo deleter mice in C57BL/6J background. PLoS ONE. (2009) 4:e8054. 10.1371/journal.pone.0008054 19956655 PMC2777316

[20] Detanico T St Clair JB Aviszus K Kirchenbaum G Guo W Wysocki LJ. Somatic mutagenesis in autoimmunity. Autoimmunity. (2013) 46:102–14. 10.3109/08916934.2012.757597 23249093 PMC3743419

[21] Love MI Huber W Anders S. Moderated estimation of fold change and dispersion for RNA-seq data with DESeq2. Genome Biol. (2014) 15:550. 10.1186/s13059-014-0550-8 25516281 PMC4302049

[22] Janeway CA Jr Travers P Walport M Shlomchik MJ Immunobiology: The Immune System in Health and Disease. 5th ed, (New York, NY: Garland Science) (2001).

[23] Lu H Lu N Weng L Yuan B Liu YJ Zhang Z. DHX15 senses double-stranded RNA in myeloid dendritic cells. J Immunol. (2014) 193:1364–72. 10.4049/jimmunol.1303322 24990078 PMC4108507

[24] Mosallanejad K Sekine Y Ishikura-Kinoshita S Kumagai K Nagano T Matsuzawa A. The DEAH-box RNA helicase DHX15 activates NF-kappaB and MAPK signaling downstream of MAVS during antiviral responses. Sci Signal. (2014) 7:ra40. 10.1126/scisignal.2004841 24782566

[25] Wang P Zhu S Yang L Cui S Pan W Jackson R. Nlrp6 regulates intestinal antiviral innate immunity. Science. (2015) 350:826–30. 10.1126/science.aab3145 26494172 PMC4927078

[26] Heng TS Painter MW Immunological Genome Project Consortium. The Immunological Genome Project: networks of gene expression in immune cells. Nat Immunol. (2008) 9:1091–4. 10.1038/ni1008-1091 18800157

[27] Chang TH Tung L Yeh FL Chen JH Chang SL. Functions of the DExD/H-box proteins in nuclear pre-mRNA splicing. Biochim Biophys Acta. (2013) 1829:764–74. 10.1016/j.bbagrm.2013.02.006 23454554

[28] Hooper C Hilliker A. Packing them up and dusting them off: RNA helicases and mRNA storage. Biochim Biophys Acta. (2013) 1829:824–34. 10.1016/j.bbagrm.2013.03.008 23528738

[29] Marintchev A. Roles of helicases in translation initiation: a mechanistic view. Biochim Biophys Acta. (2013) 1829:799–809. 10.1016/j.bbagrm.2013.01.005 23337854 PMC3640703

[30] Lee PP Fitzpatrick DR Beard C Jessup HK Lehar S Makar KW. A critical role for Dnmt1 and DNA methylation in T cell development, function, and survival. Immunity. (2001) 15:763–74. 10.1016/S1074-7613(01)00227-8 11728338

[31] Neckers LM Cossman J. Transferrin receptor induction in mitogen-stimulated human T lymphocytes is required for DNA synthesis and cell division and is regulated by interleukin 2. Proc Natl Acad Sci USA. (1983) 80:3494–8. 10.1073/pnas.80.11.3494 6304712 PMC394071

[32] Ned RM Swat W Andrews NC. Transferrin receptor 1 is differentially required in lymphocyte development. Blood. (2003) 102:3711–8. 10.1182/blood-2003-04-1086 12881306

[33] Mills DM Stolpa JC Cambier JC. Cognate B cell signaling via MHC class II: differential regulation of B cell antigen receptor and MHC class II/Ig-alpha beta signaling by CD22. J Immunol. (2004) 172:195–201. 10.4049/jimmunol.172.1.195 14688326

[34] Nakano-Yokomizo T Tahara-Hanaoka S Nakahashi-Oda C Nabekura T Tchao NK Kadosaki M. The immunoreceptor adapter protein DAP12 suppresses B lymphocyte-driven adaptive immune responses. J Exp Med. (2011) 208:1661–71. 10.1084/jem.20101623 21727189 PMC3149228

[35] Jabara HH Boyden SE Chou J Ramesh N Massaad MJ Benson H. A missense mutation in TFRC, encoding transferrin receptor 1, causes combined immunodeficiency. Nat Genet. (2016) 48:74–8. 10.1038/ng.3465 26642240 PMC4696875

[36] Griffiths GM Berek C Kaartinen M Milstein C. Somatic mutation and the maturation of immune response to 2-phenyl oxazolone. Nature. (1984) 312:271–5. 10.1038/312271a0 6504141

[37] Lanzavecchia A. Antigen-specific interaction between T and B cells. Nature. (1985) 314:537–9. 10.1038/314537a0 3157869

[38] Almaden JV Tsui R Liu YC Birnbaum H Shokhirev MN Ngo KA. A pathway switch directs BAFF signaling to distinct NFkappaB transcription factors in maturing and proliferating B cells. Cell Rep. (2014) 9:2098–111. 10.1016/j.celrep.2014.11.024 25497099 PMC4889572

[39] Arezes J Costa M Vieira I Dias V Kong XL Fernandes R. Non-transferrin-bound iron (NTBI) uptake by T lymphocytes: evidence for the selective acquisition of oligomeric ferric citrate species. PLoS ONE. (2013) 8:e79870. 10.1371/journal.pone.0079870 24278199 PMC3836815

[40] Inesta-Vaquera F Chaugule VK Galloway A Chandler L Rojas-Fernandez A Weidlich S. DHX15 regulates CMTR1-dependent gene expression and cell proliferation. Life Sci Alliance. (2018) 1:e201800092. 10.26508/lsa.201800092 30079402 PMC6071836

