# Supplementary material for: Co-expression Networks Identify DHX15 RNA Helicase as a B Cell Regulatory Factor
Source: Front Immunol. 2019 Dec 10;10:2903. doi: 10.3389/fimmu.2019.02903 (PMC6915936; doi:10.3389/fimmu.2019.02903)
Supplement: Supplementary file 1 [file Data_Sheet_1.PDF]

Supplementary Fig 1A

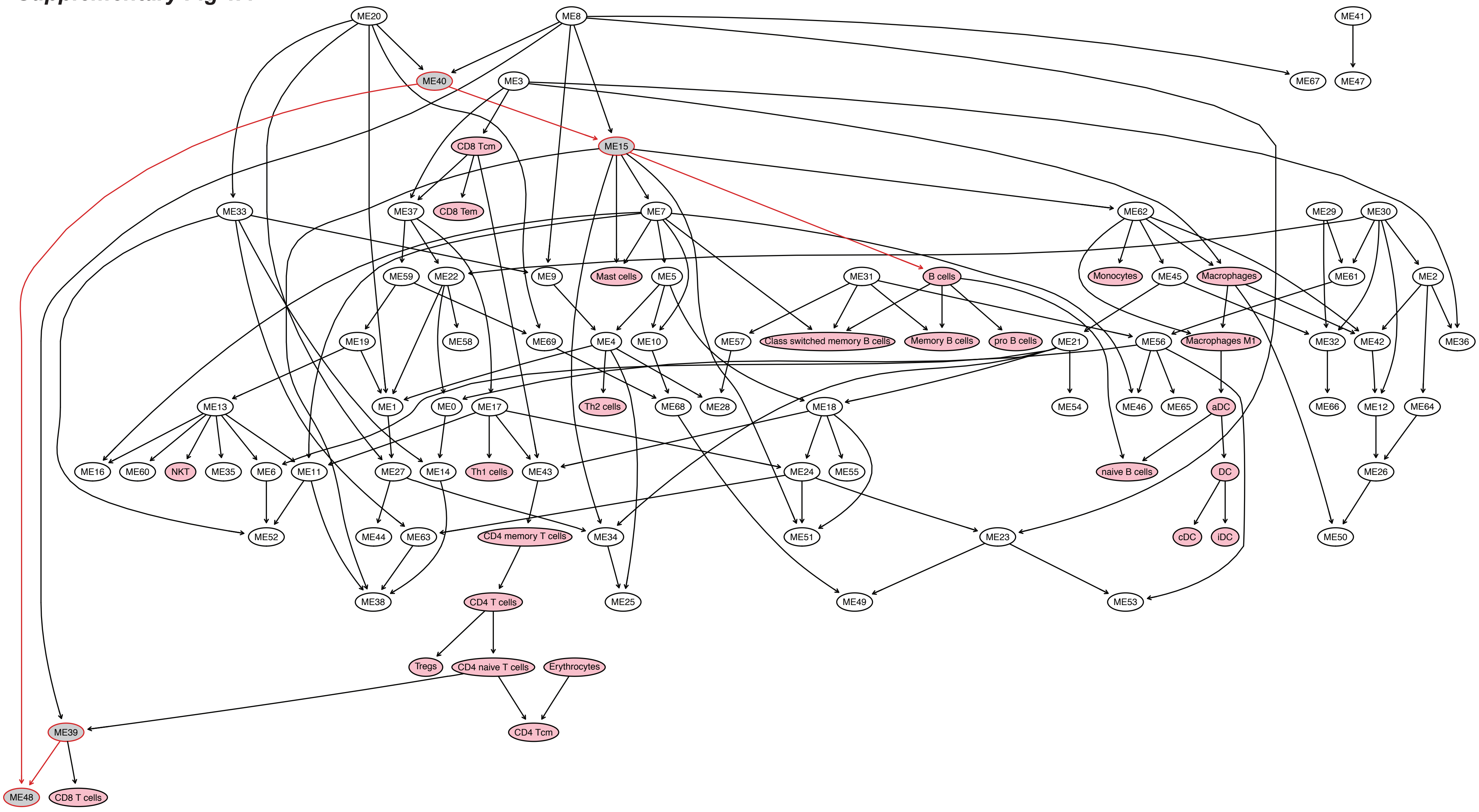

Supplementary Fig 1B

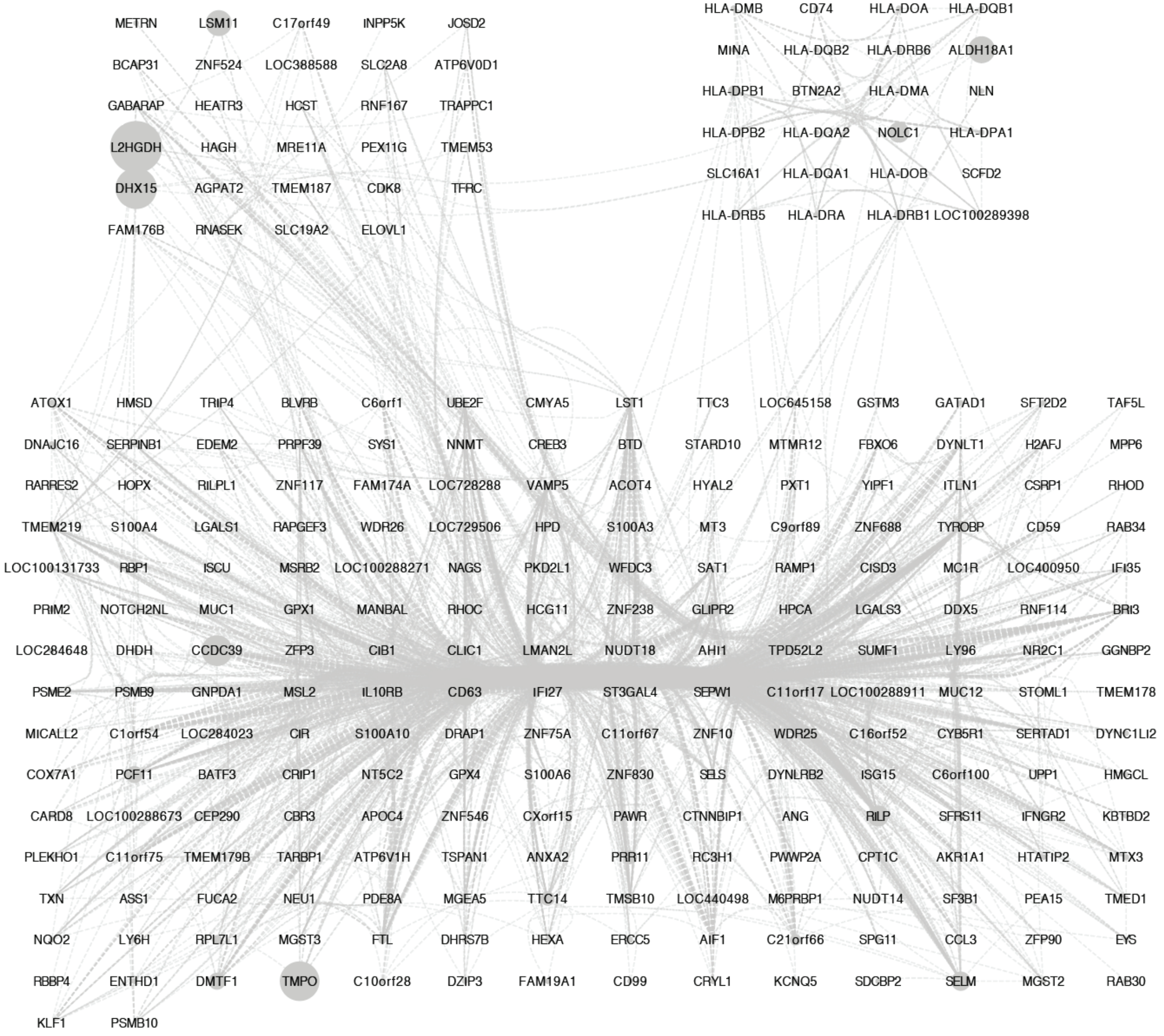

Supplementary Fig. 1:

Cell signature and complete modular immune-network. ***A,***RNAseq data from Schimitz et al was used in our GMIC pipeline to create a modular immune-network. Immune cell signatures are represented as red filled boxes. Pathway and modules (ME) showed in Figure 1B are represented by the red arrows and grey filled boxes. Arrow directions suggests causality. ***B,*** Expanded version of Figure 1C. Nodes size represent betweenness-centrality calculated by Cytoscape from the depicted directed network using the WGCNA results.

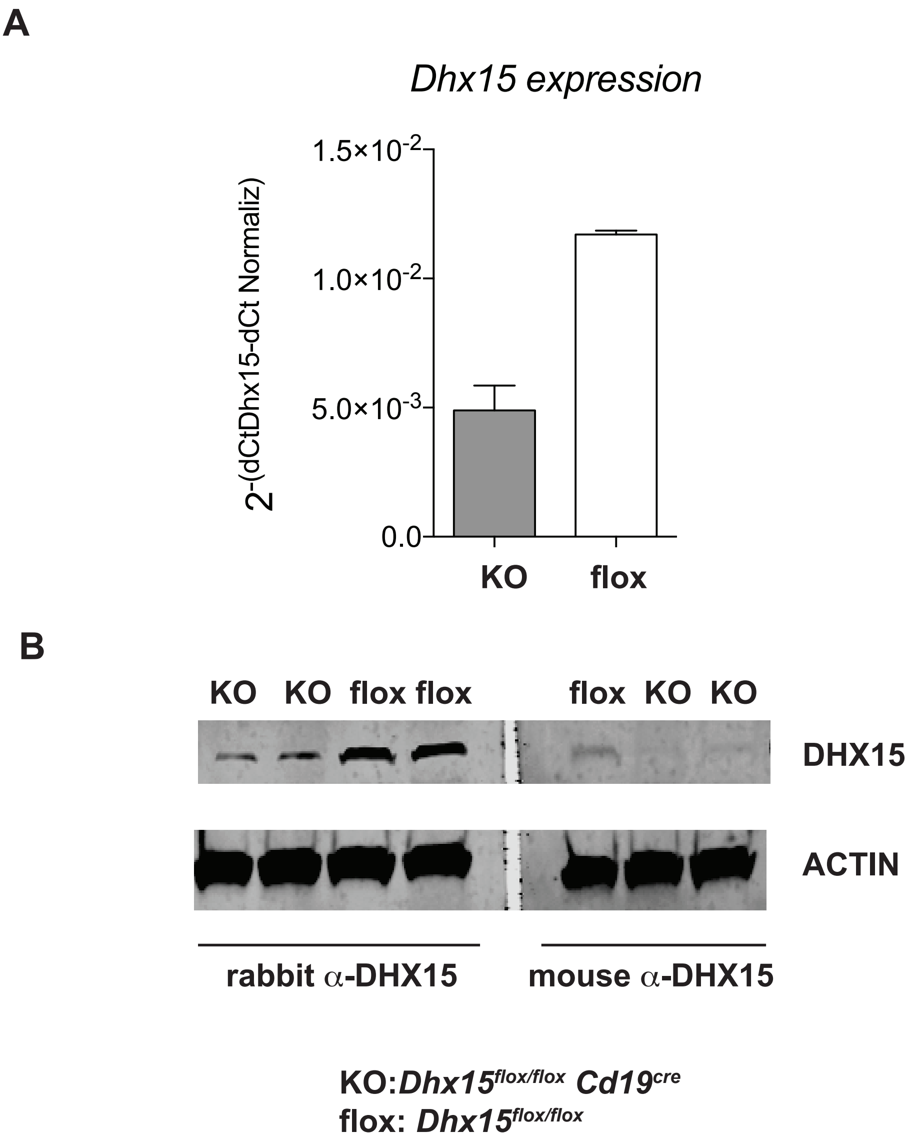

Supplementary Fig. 2:
DHX15 deletion in conditional *Dhx15*$^{flox/flox}$ *Cd19*^{cre}.
A, RT-qPCR from purified B cells (86-92% purity) for expression of *Dhx15* normalized by the expression of *ActinB*.
B, Western blot for DHX15 and Actin on purified B cells (88-92% purity), using two different anti-DHX15 Abs. Left panel, a rabbit anti-DHX15 A300-390A from Bethyl Laboratories Inc., and right panel an anti-DDX15 clone E6 mouse monoclonal from Santa Cruz Biotechnology.
DHX15 protein expression was reduced approximately 84% on KO B cells (left panel).

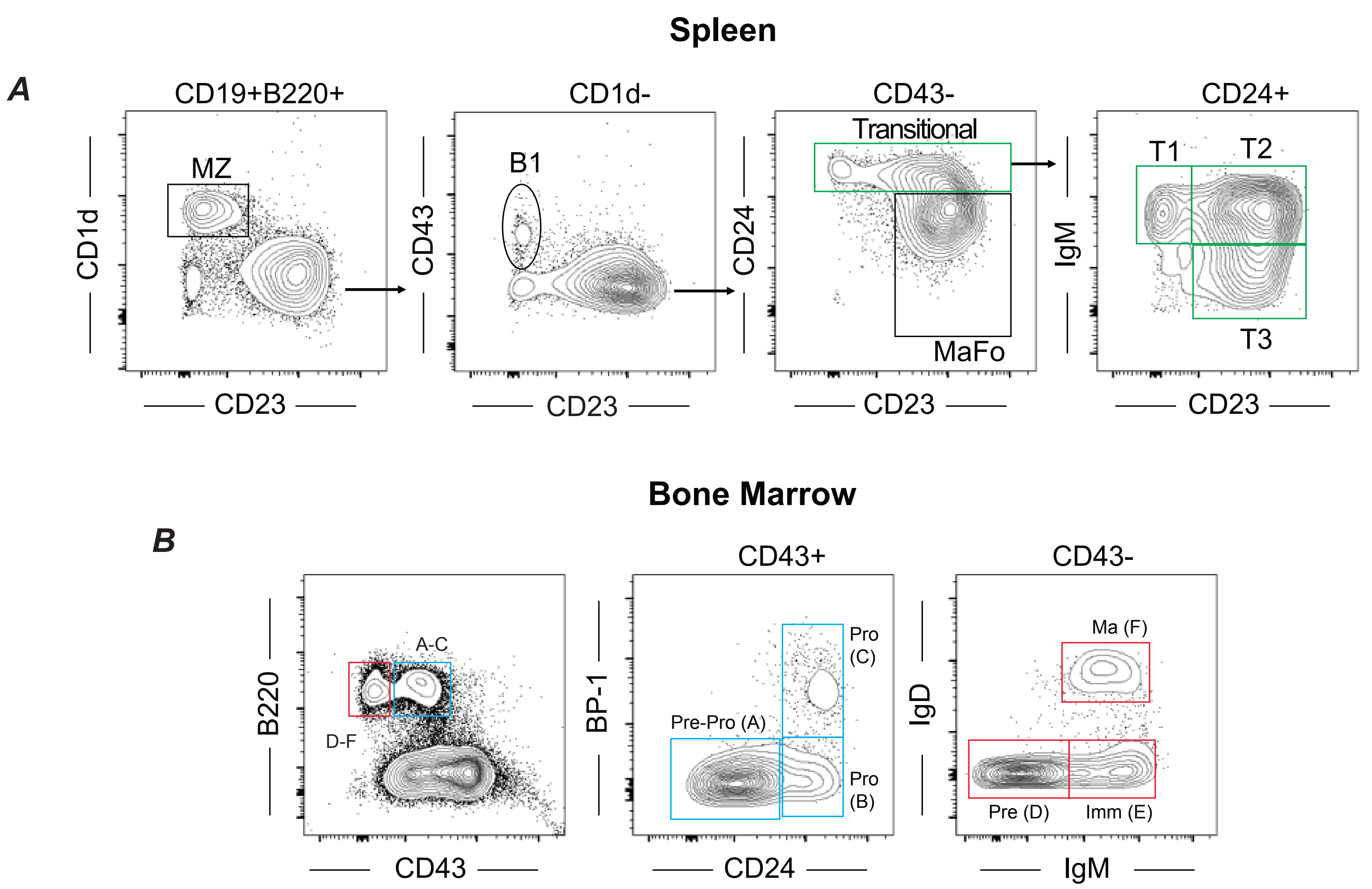

Supplementary Fig 3:
B cell gating strategy. ***A,*** Splenic B cell subtypes. MZ (Marginal Zone) and MaFo (Mature Follicular). Trasitional stages (right far panel) were also validated by IgD stain (data not shown).
B, Bone marrow gating scheme. B cell development was determined using Hardy's fractions definition (A-F fractions).

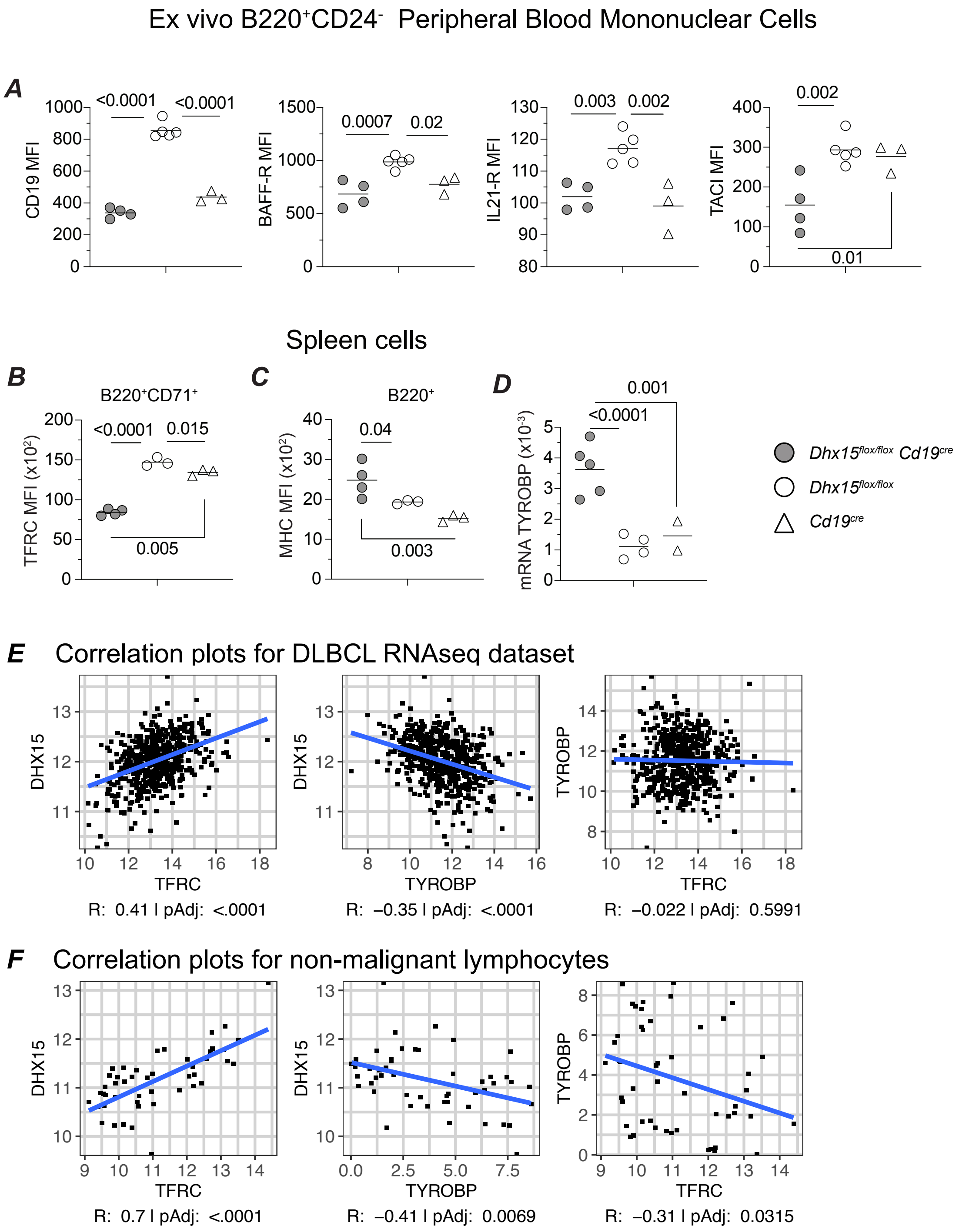

Supplementary Fig 4

Expression of potential DHX15 target genes.

Some of the differentially expressed genes observed in Figure 4 were validated *ex vivo* by flow cytometry or RT-qPCR. ***A,*** Graphs represent the Median Fluorescence Intensity (MFI) of the indicated proteins on *ex vivo* B220$^+$CD24$^-$PBMCs. ***B-C,*** Spleen B cells were stimulated with α-IgM and α-CD40 for 48h.***D,***mRNA expression of purified CD43$^-$ B cells normalized to Actin. ***E,*** Correlation plots for the DLBCL RNAseq dataset used in Figure 1. ***F,*** Correlation plots for non-malignant lymphocytes. RNAseq expression data for non-malignant lymphocytes was obtained from https://amp.pharm.mssm.edu/archs4 and normalized using DESeq2. For sample GEO accession numbers, annotations, and expression values see Supplementary Table 3. Filled circles *Dhx15$^{flox/flox}$ Cd19^{cre}*, opened circles *Dhx15$^{flox/flox}$* and opened triangles *Cd19^{cre}* mice. Each symbol represents an individual mouse. Animals were 8-14 weeks of age, and from both sexes. Statistical analysis was performed with R studio using the multiple linear regression funtion and the following equation: rank(MFI)~Genotype. Only P values smaller than 0.05 were reported.
